# Glycolytic reprogramming and immune responses in macrophages: a crosstalk driven by bacterial infection

**DOI:** 10.3389/fimmu.2026.1783266

**Published:** 2026-04-10

**Authors:** Wei-ren Wang, Lin Yan, Chuan-ying Zhao, Cong-cong He, Xing-Hua Gao

**Affiliations:** 1 Department of Dermatology, The First Hospital of China Medical University, Shenyang, China; 2 Key Laboratory of Immunodermatology, Ministry of Education and NHC, National Joint Engineering Research Center for Theranostics of Immunological Skin Diseases, Shenyang, China

**Keywords:** bacterial infection, glycolytic reprogramming, immunometabolism, macrophage polarization, metabolic signaling

## Abstract

Macrophage glycolytic reprogramming during bacterial infection is a recognized metabolic shift with profound yet incompletely defined immunological consequences. This review delineates how this metabolic remodeling extends beyond energy provision to function as an integral immunoregulatory platform. We systematically examine the dual roles of key metabolic components, including the conformational dynamics of pyruvate kinase M2 that couple metabolic flux with inflammatory gene transcription, and the NAD^+^/NADH ratio that balances inflammasome activation against interferon responses. The review further explores how metabolites like lactate, succinate, and itaconate mediate immunomodulation through novel post-translational modifications, including histone lactylation and protein succinylation. Crucially, we analyze how diverse bacterial pathogens such as Salmonella and Mycobacterium tuberculosis exploit these metabolic networks for immune evasion. By integrating recent advances in host immunometabolism with bacterial pathogenesis, this work not only deciphers critical molecular dialogues at the host-pathogen interface but also identifies novel targetable pathways, offering a conceptual framework for developing innovative therapeutic strategies against persistent and antibiotic-resistant infections.

## 1 Introduction

Macrophages, as central effector cells of the innate immune system, recognize pathogen-associated molecular patterns (PAMPs) through pattern recognition receptors (PRRs), including Toll-like receptor 4 (TLR4) and NOD-like receptors (NLRs). This recognition triggers phagocytosis and activates inflammatory cascades, constituting the host’s first line of defense against bacterial invasion (1). Their functional plasticity allows differentiation into pro-inflammatory (M1) or anti-inflammatory (M2) subsets within the infection microenvironment (2). This polarization is initially triggered by the recognition of PAMPs, such as through the TLR4/NF-κB signaling pathway, which induces M1 polarization, and is subsequently reinforced and maintained by coupled glycolytic reprogramming (3, 4). M1 macrophages directly eliminate pathogens via the secretion of pro-inflammatory mediators such as interleukin-1β (IL-1β) and tumor necrosis factor-α (TNF-α), whereas M2 macrophages promote tissue repair through anti-inflammatory factors including interleukin-10 (IL-10) and transforming growth factor-β (TGF-β) (2).

As a fundamental metabolic pathway, glycolysis rapidly converts glucose into pyruvate, providing a net gain of ATP and key metabolic intermediates to power the antimicrobial functions of macrophages during bacterial infection. This is achieved through a metabolic shift from oxidative phosphorylation (OXPHOS) to aerobic glycolysis, a phenomenon reminiscent of the “Warburg effect”. This reprogramming is characterized by the upregulation of key glycolytic enzymes, including glucose transporter 1 (GLUT1), hexokinase 2 (HK2), pyruvate kinase M2 (PKM2), and lactate dehydrogenase A (LDHA), alongside the suppression of OXPHOS. The ensuing glycolytic flux supports the rapid generation of ATP and nicotinamide adenine dinucleotide phosphate (NADPH), which collectively provide the necessary energy and reducing equivalents for the burst secretion of pro-inflammatory factors and enhanced phagocytic activity, thereby establishing a robust host defense (5–8).

Macrophage metabolic remodeling and immune responses engage in complex bidirectional regulation (1). On one hand, glycolytic metabolism provides energy and molecular substrates that support immune activation. On the other hand, pathogens can exploit host metabolic intermediates such as succinate and itaconate to modulate infection outcomes in their favor (9, 10).This immunometabolic crosstalk is finely regulated by signaling pathways including mammalian target of rapamycin (mTOR), hypoxia-inducible factor-1α (HIF-1α), and AMP-activated protein kinase (AMPK). HIF-1α promotes aerobic glycolysis by upregulating glycolytic enzymes and inhibiting pyruvate dehydrogenase (PDH) activity (11), whereas AMPK enhances fatty acid oxidation (FAO) by suppressing mTOR complex 1 (mTORC1) signaling, facilitating the transition from M1 to M2 phenotypes (12). Moreover, metabolic intermediates themselves serve as signaling molecules: lactate promotes M2 gene expression (such as arginase 1 [Arg1] and IL-10) via histone lactylation (13), and succinate enhances pro-inflammatory gene transcription through succinylation (14).

Although current studies have outlined the immunoregulatory framework of metabolic reprogramming, several key aspects in the context of bacterial infection require further elucidation (15). These include the metabolic specificity of macrophages in response to different pathogens, the trade-offs of metabolic reprogramming for intracellular bacterial survival, and the spatiotemporal precision of metabolic interventions (16). This review systematically examines the mechanisms of bacterial infection-driven glycolytic remodeling in macrophages, integrates the interactions within the HIF-1α, nuclear factor-kappa B (NF-κB), phosphoinositide 3-kinase/protein kinase B/mammalian target of rapamycin (PI3K/Akt/mTOR), and AMPK signaling networks, elucidates the epigenetic and immunoregulatory functions of metabolic intermediates, and explores precise therapeutic strategies targeting the metabolism-immune interface, aiming to provide new perspectives for immune intervention in infectious diseases.

## 2 Mechanisms of glycolytic remodeling in macrophages during bacterial infection

Glycolysis, a core energy metabolic pathway, involves the stepwise conversion of glucose to pyruvate via rate-limiting enzymes including hexokinase (HK), phosphofructokinase (PFK), and pyruvate kinase (PK) (17). Under basal conditions, glucose uptake via GLUT1 is followed by HK-mediated phosphorylation to glucose-6-phosphate (G6P), with PFK and PK sequentially catalyzing metabolic flux toward pyruvate (18, 19). In the presence of oxygen, pyruvate is converted to acetyl-coenzyme A (acetyl-CoA) by PDH for entry into the tricarboxylic acid (TCA) cycle, yielding up to 36 ATP molecules per glucose through OXPHOS, which far exceeding the net yield of 2 ATP from glycolysis alone (3, 20). The dynamic reprogramming of rate-limiting enzymes constitutes the core of metabolic adaptation in macrophages during bacterial infection. PAMPs such as lipopolysaccharide (LPS) markedly upregulate HK expression to enhance glucose phosphorylation (21, 22),while LDHA converts pyruvate into lactate to sustain nicotinamide adenine dinucleotide (NAD^+^) regeneration (23). Concurrent inhibition of PDH diverts metabolic flux toward lactate production, collectively establishing a classical “aerobic glycolysis” phenotype. This metabolic hijacking driven by activation of HK and PK coupled with suppression of oxidative phosphorylation, shifts cellular metabolism from an energy efficient mode toward a rapid-response state. Such rewiring, mediated by GLUT1-dependent glucose uptake, coordinated activation of HK and LDHA, and suppression of mitochondrial function (24), not only supplies ATP, NADPH, and biosynthetic precursors essential for antimicrobial defense but also enables glycolytic intermediates such as lactate and succinate to act as immunomodulatory signals that amplify inflammatory responses. Thus, glycolysis transcends its conventional role in energy supply to emerge as a pivotal immunometabolic hub.

### 2.1 Key molecular regulators of glycolytic remodeling

During bacterial infection, an interactive immunometabolic network is constructed by core glycolytic remodelers, including NAD^+^/NADH, PKM2, and GLUT1/sirtuin 5 (SIRT5), which collectively regulate energy flux, redox balance, and inflammatory signaling to drive macrophage functional remodeling.

#### 2.1.1 NAD^+^/NADH ratio: a bridge linking redox balance and inflammation

Bacterial infection-induced aerobic glycolysis significantly lowers the intracellular NAD^+^/NADH ratio in macrophages through two primary mechanisms: glycolytic activation consumes NAD^+^ as a cofactor, while impaired mitochondrial electron transport chain function particularly LPS-mediated suppression of Electron Transport Chain Complex I (NADH:ubiquinone oxidoreductase) activity that impedes NADH oxidation leads to NADH accumulation (25–27). This redox imbalance profoundly influences immune responses, as demonstrated in *Salmonella enterica* serovar Typhimurium infection models where pathogen-induced expression of key glycolytic enzymes (HK2, LDHA) and inhibition of mitochondrial Complex I function drive abnormal NADH accumulation and significantly reduce the NAD^+^/NADH ratio (28). The resulting metabolic perturbation promotes NLRP3 inflammasome assembly and IL-1βmaturation/release through activation of the HIF-1α-dependent glycolysis-succinate axis, while simultaneously suppressing mitochondrial antiviral-signaling protein interferon regulatory factor 3 (MAVS-IRF3) mediated type I interferon (IFN-β) production.

Concurrent with these changes, TCA cycle disruption causes aberrant succinate accumulation, which amplifies inflammatory responses via HIF-1α-dependent pathways (29). Impaired NAD^+^ synthesis further exacerbates the buildup of pro-inflammatory intermediates including succinate and malate (30), creating a self-reinforcing “metabolism-inflammation” positive feedback loop.

Experimental evidence confirms the central role of this metabolic switch: restoring the NAD^+^/NADH ratio through supplementation of NAD^+^ precursors or overexpression of NAD^+^ synthase significantly curbs pro-inflammatory cytokine secretion while enhancing stimulator of interferon genes (STING)-dependent IFN-β expression. The critical role of the NAD^+^/NADH ratio is further validated by interventional approaches: artificially disturbing this ratio through inhibition of glycolysis or Complex I is sufficient to reprogram immune responses, thereby establishing its function as a core metabolic switch that balances pro-inflammatory and antiviral pathways. This regulatory mechanism, observed primarily in *in vitro* and murine models (30, 31), may extend beyond specific pathogens. These findings collectively suggest mitochondrial redox status as a critical node for immune regulation, although its role in human infections requires further validation (32). Although the precise molecular mechanisms underlying NADH’s direct regulation of inflammation require further elucidation, therapeutic strategies targeting this axis show promise. Exogenous modulation of NADH synthesis or degradation enzymes through overexpression of NAD kinase (NADK) or inhibition of NADPH oxidase (NOX) activity can mitigate excessive inflammation in disease by directionally regulating the NAD^+^/NADH ratio, highlighting its potential as a therapeutic target for infectious diseases (33, 34).

#### 2.1.2 PKM2: a dual regulator of glycolysis and inflammatory burst

Pyruvate kinase (PK), a critical rate-limiting enzyme in glycolysis, catalyzes the conversion of phosphoenolpyruvate to pyruvate. The M2 isoform (PKM2) dynamically regulates macrophage immunometabolic balance through conformational switching between tetramers and dimers. The tetrameric state exhibits high catalytic activity, directing pyruvate toward acetyl-CoA formation and mitochondrial OXPHOS to support energy homeostasis. In contrast, The dimeric conformation displays reduced enzymatic activity, favoring pyruvate reduction to lactate and facilitating a Warburg-like metabolic state. *In vitro* studies have demonstrated that LPS stimulation can promote PKM2 transition into dimers and lead to its nuclear translocation in macrophages (35–37). Animal models further suggest that myeloid-specific PKM2 deletion attenuates inflammasome activation (38).The pathological significance of this dimerization and nuclear translocation is substantiated by genetic evidence, as myeloid-specific PKM2 knockout in murine models has been shown to suppress NLRP3 and absent in melanoma 2 (AIM2) inflammasome activation and significantly reduces mortality in both endotoxemia and polymicrobial sepsis models (38).This conformational switching is biochemically regulated by post-translational modifications. Phosphorylation of PKM2 stabilizes the dimeric form, reinforcing the Warburg phenotype by preventing tetramer assembly (39, 40). Inhibition of this phosphorylation suppresses dimer formation and nuclear translocation, thereby attenuating downstream inflammatory signaling.

Inside the nucleus, PKM2 dimers function as transcriptional co-activators. They associate with HIF-1α and bind directly to the IL-1β promoter to enhance its transcription (41, 41). Concurrently, nuclear PKM2 amplifies pro-inflammatory signaling by promoting signal transducer and activator of transcription 3 (STAT3) phosphorylation (42). These actions establish a self-sustaining “metabolic remodeling inflammatory burst” positive feedback loop. The critical role of nuclear PKM2 in driving pathology is further highlighted by the action of the natural compound celastrol, which inhibits PKM2 nuclear translocation by targeting specific cysteine residues, significantly mitigating cytokine storms (TNF-α, IL-6, IL-1β) and multi-organ injury in sepsis models (43).

The interplay between PKM2 and HIF-1α forms a reinforcing regulatory circuit. HIF-1α, upregulated through TLR4/NF-κB signaling upon LPS stimulation, transcriptionally enhances PKM2 expression, thereby increasing glycolytic capacity (41, 44). In turn, nuclear PKM2 dimers act as co-activators for HIF-1α, binding its transactivation domain to augment the expression of glycolytic genes such as LDHA and pro-inflammatory mediators including IL-1β and high mobility group box 1 (HMGB1) (45). This reciprocal regulation, observed during Salmonella infection, not only sustains Warburg metabolism to energize immune responses but also amplifies IL-1β and HMGB1 release, strengthening antimicrobial defense (36).

Beyond its pro-inflammatory role, PKM2 orchestrates a protective metabolic program essential for inflammation resolution, with its tetrameric conformation serving as a critical regulatory node. The small-molecule activator TEPP-46 demonstrates multi-faceted immunometabolic regulation by stabilizing PKM2 tetramers, which not only augments glycolytic flux but also redirects glucose metabolism away from toxic intermediate accumulation (e.g., sorbitol, methylglyoxal, and diacylglycerol) toward mitochondrial energy production. This stabilization suppresses LPS-induced pathological glycolysis and succinate accumulation, thereby blocking HIF-1α binding to the IL-1β promoter and subsequent IL-1β production while promoting anti-inflammatory IL-10 secretion (36). These coordinated metabolic changes facilitate mitochondrial functional recovery through upregulation of peroxisome proliferator-activated receptor gamma coactivator 1-alpha (PGC-1α) and optic atrophy 1 (OPA1), enhancing mitochondrial biogenesis and fusion (46). The resulting restoration of oxidative phosphorylation couples with macrophage phenotypic switching, where PKM2 activation attenuates pro-inflammatory M1 polarization while promoting reparative M2 differentiation (36). However, the therapeutic landscape requires careful navigation, as TEPP-46 may potentially enhance RNA-dependent protein kinase (PKR) phosphorylation, which could activate the NLRP3 inflammasome and increase inflammatory cytokine release, illustrating the challenge of fine-tuning immunometabolism without disrupting host homeostasis.

Collectively, these findings establish PKM2 as a pivotal metabolic-immune integrator that balances antimicrobial defense against inflammatory damage, providing a theoretical foundation for spatiotemporally precise therapeutic strategies in inflammatory diseases such as sepsis.

#### 2.1.3 GLUT1 and SIRT5: coordinated regulation of glucose uptake and metabolic homeostasis

During bacterial infection, marked upregulation of the core glucose transporter GLUT1 serves as a critical initiating point for metabolic reprogramming in macrophages, substantially enhancing glucose uptake to fuel glycolytic flux (47, 48). This GLUT1-driven glucose influx initiates a broader signaling network that extends beyond energy production. It stimulates insulin secretion, and the resulting insulin engages macrophage insulin receptors (InsR), activating downstream Akt and c-Jun N-terminal kinase (JNK) phosphorylation. This signaling cascade further promotes reactive oxygen species (ROS) generation and IL-1β release, establishing a self-amplifying “glucose-insulin-IL-1β” positive feedback loop that intensifies antibacterial responses. Within this context, GLUT1 functions not merely as a nutrient gatekeeper but as a pivotal initiator of an integrated immunometabolic circuit.

The SIRT5 operates as a key modulator within this GLUT1-initiated network, exerting bidirectional control over inflammatory outcomes. SIRT5 deficiency exacerbates IL-1β production (49), potentially through amplification of the pro-inflammatory arm of insulin signaling and disruption of mitochondrial metabolic homeostasis. Notably, SIRT5 also plays a determinative role in M2 macrophage polarization, where its elevated expression in eosinophilic nasal polyps correlates positively with M2 markers and facilitates alternative activation by promoting glutaminolysis (50). The inherent capacity of SIRT5 to suppress IL-1β suggests a protective role against excessive inflammation, while its established role in promoting M2 macrophage polarization reveals complex context-dependent regulation. Concurrently, SIRT5 maintains metabolic balance through regulation of enzyme activity, as evidenced in clear cell renal cell carcinoma where it desuccinylates PDH E1 subunit alpha 1 (PDHA1) at K351 to restrain the Warburg effect and sustain TCA cycle functionality (51). Pathogens may potentially subvert host defense by targeting SIRT5 activity, thereby co-opting insulin-related pathways to remodel immune responses (52). Collectively, these interactions position GLUT1-mediated glucose metabolism as the central hub, with insulin signaling and the SIRT5 regulatory node acting as critical modulators, defining a key molecular interface governing metabolic-immune equilibrium during infection.

### 2.2 Signaling network governing glycolytic remodeling

Macrophage glycolytic remodeling represents a sophisticated regulatory paradigm where multiple signaling pathways converge to form an integrated network. This complex process is driven by the synergistic action of multidimensional signaling pathways, with HIF-1α, AMPK, PI3K/Akt/mTOR, and NF-κB constituting the core regulatory framework. These pathways dynamically balance pro- and anti-inflammatory responses through their interconnected actions, coordinating metabolic adaptation with immune function by integrating the regulation of metabolic enzyme activity, modulating mitochondrial function, and fine-tuning immune signal amplification. The four key molecular hubs operate not in isolation but through dynamic interconnections that enable macrophages to precisely adjust their metabolic state according to infection status and energy demands, establishing a fundamental mechanism that links cellular metabolism with immunological outcomes in the antibacterial response.

At the foundation of this network, AMPK serves as the primary energy sensor that initiates metabolic adaptation. As a central regulator of cellular energy homeostasis, AMPK responds to metabolic stress by sensing fluctuations in intracellular ATP levels. Upon activation, it promotes catabolic pathways, such as glucose uptake, glycolysis, and FAO, to facilitate energy production, while simultaneously inhibiting anabolic processes including fatty acid synthesis and gluconeogenesis to reduce energy consumption (53, 54). When activated under conditions of metabolic stress, AMPK enhances glycolytic capacity through multiple mechanisms: it promotes glucose uptake by facilitating GLUT1 translocation to the plasma membrane (55) and fine-tunes glycolytic flux by regulating hexokinase activity (56). Beyond its metabolic functions, AMPK exerts broad regulatory control over inflammatory signaling. It directly constrains the Warburg effect by suppressing HIF-1α transcriptional activity (57, 58) and limits excessive inflammation by phosphorylating NF-κB subunits to restrict their nuclear translocation (59). The AMPK-mTOR axis represents another critical regulatory interface, where AMPK activation inhibits mTORC1 signaling through the tuberous sclerosis complex (TSC), effectively balancing catabolic and anabolic processes according to cellular energy status (60, 61). Recent findings further expand AMPK’s functional repertoire, demonstrating that Fms related receptor tyrosine kinase 4 (FLT4)-mediated phosphorylation of AMPKα at specific tyrosine residues links glycolytic reprogramming with autophagy-dependent bacterial clearance and inflammasome activation (12), suggesting that AMPK functions as a key integrator of metabolic and immune signals in preclinical models.

Building upon this foundation, HIF-1α emerges as the principal executor of glycolytic commitment, integrating signals from upstream pathways such as NF-κB, which directly enhances its transcription (62, 63), and mTORC1 downstream of PI3K/Akt signaling, which further potentiates HIF-1α activity (64). Once stabilized, HIF-1α orchestrates a comprehensive metabolic shift by transcriptionally activating key glycolytic enzymes including HK2, PKM2, LDHA, and pyruvate dehydrogenase kinase 1 (PDK1) (65–69). This enzymatic reprogramming redirects carbon flux from mitochondrial oxidation toward glycolysis, with LDHA playing the dual role of catalyzing pyruvate-to-lactate conversion while regenerating NAD^+^ to sustain glycolytic flux (70). The metabolic consequences extend beyond energy production, as TCA cycle interruption leads to succinate accumulation, which further stabilizes HIF-1α by inhibiting prolyl hydroxylases (PHDs), creating a powerful positive feedback loop that amplifies IL-1β transcription and inflammatory responses (29). The HIF-1α-PDK1 axis further reinforces this metabolic state by inhibiting pyruvate entry into mitochondria, suppressing OXPHOS while enhancing glycolytic dependency (71).

The inflammatory and metabolic networks achieve sophisticated integration through crosstalk between NF-κB and PI3K/Akt/mTOR signaling. Bacterial components like LPS activate NF-κB through TLR4 receptors, driving the production of pro-inflammatory cytokines including TNF-α and IL-6 that constitute essential host defense mechanisms (72). However, this inflammatory response is precisely calibrated by the PI3K/Akt/mTOR pathway. PI3K-mediated Akt phosphorylation leads to mTORC1 activation, which subsequently initiates a negative feedback loop that attenuates PI3K/Akt signaling (73–77), preventing NF-κB hyperactivation and maintaining inflammatory homeostasis (78).

The NF-κB-HIF-1α interface represents another critical integration point that exemplifies the network’s complexity. While NF-κB enhances HIF-1α transcription under inflammatory conditions, HIF-1α reciprocally limits excessive inflammation by inhibiting NF-κB transcriptional activity (79). This bidirectional regulation ensures that metabolic reprogramming supports rather than exacerbates inflammatory responses. Meanwhile, mTORC1 promotes the metabolic transition to glycolysis by activating HIF-1α and its downstream targets including LDHA and PKM2 (37, 64), creating a coordinated response that aligns energy metabolism with immune function.

What emerges from this analysis is a sophisticated regulatory architecture characterized by multiple feedback and feedforward loops. AMPK activation initiates metabolic adaptation while constraining inflammatory excess through direct effects on both HIF-1α and NF-κB. HIF-1α then executes glycolytic commitment, with its activity modulated by both upstream inflammatory signals (NF-κB) and metabolic regulators (mTORC1). The PI3K/Akt/mTOR pathway integrates these signals, providing both positive drive for metabolic reprogramming and negative feedback to prevent inflammatory excess. NF-κB serves as the inflammatory engine while being progressively constrained by both HIF-1α and mTOR-mediated feedback mechanisms.

**Figure 1:**
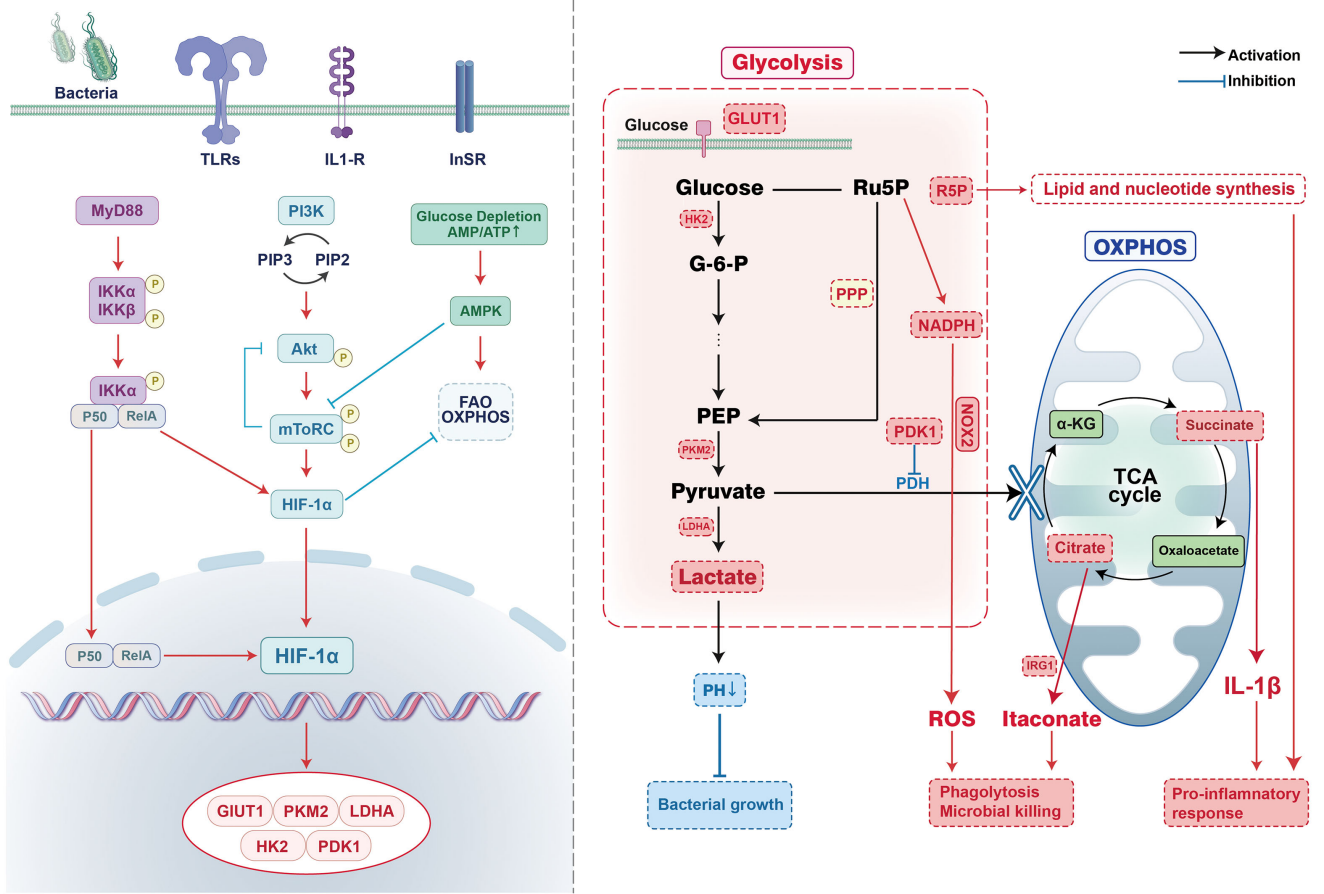
Signaling pathways and processes of bacterial infection-driven glycolytic remodeling in macrophages. Upon bacterial recognition, an integrated signaling network converges to stabilize the master transcriptional regulator HIF-1α. This in turn initiates a profound metabolic shift by upregulating key glycolytic enzymes including GLUT1, HK2, PKM2, LDHA, and PDK1 collectively enhancing glucose uptake and flux while suppressing mitochondrial oxidation. The resulting metabolic rewiring directly fuels the antimicrobial response: lactate production acidifies the microenvironment; glycolytic and PPP-derived NADPH enables ROS generation via NOX2; TCA cycle interruption leads to accumulation of immunomodulatory succinate, which reinforces HIF-1α stability and IL-1β production; and IRG1-dependent itaconate synthesis provides direct antibacterial activity. Thus, this core immunometabolic circuit translates pathogen detection into a metabolically empowered effector response.

This intricate network enables macrophages to dynamically adjust their metabolic and inflammatory states according to infection status, balancing the urgent energy demands of bacterial clearance against the potential damage of uncontrolled inflammation. A growing understanding of these interconnected pathways, primarily derived from *in vitro* and animal studies, is beginning to reveal the sophisticated mechanisms underlying immunometabolic coordination. This complexity underscores the need for caution, suggesting that future therapeutic strategies must consider the network’s architecture rather than targeting isolated components, pending further validation in human systems (**Figure 1**).

## 3 Immunological roles of glycolytic remodeling in macrophages

The dynamic reprogramming of glucose metabolism from oxidative phosphorylation to aerobic glycolysis represents more than an energy shift, it constitutes a fundamental mechanism that directly instructs macrophage immune function. This metabolic transition, characterized by suppressed mitochondrial respiration and accelerated glycolytic flux even under normoxic conditions, enables macrophages to rapidly generate ATP and critical biosynthetic precursors while simultaneously producing immunomodulatory metabolites that shape immune responses (28, 80). The LPS-induced “Warburg effect” preferentially directs glucose metabolism through aerobic glycolysis under normoxic conditions, leading to substantial lactate production (81, 82), The immunological significance of this metabolic rewiring extends beyond energy provision. During infection, the glycolytic program supports burst cytokine secretion and antibacterial defense in M1-polarized macrophages, while the dynamic regulation of metabolic intermediates particularly through the NAD+/NADH ratio fine-tunes the balance between pro-inflammatory and anti-inflammatory signaling (83, 84). This metabolic-immune crosstalk evolves throughout the infection timeline, with glycolytic metabolism dominating the acute phase for antimicrobial functions and OXPHOS gradually resuming during resolution to promote tissue repair (85).

This bidirectional metabolic-immune network exhibits sophisticated regulatory properties, where metabolic status regulates immune response intensity while immune reactions reshape the metabolic microenvironment (86, 87). Integrated multi-omics approaches have further elucidated how metabolic characteristics under different activation states associate with gene expression patterns, identifying tryptophan metabolism and Fc receptor-mediated glycolysis as key nodes in functional remodeling (88). The direct impact of macrophage metabolic state on immune efficiency is particularly evident in severe infections such as sepsis, where it significantly influences patient prognosis (89). The following sections systematically examine how glycolytic remodeling orchestrates immune regulation through metabolite signaling, pathogen hijacking strategies, M1/M2 polarization coupling, and therapeutic interventions.

### 3.1 Immunomodulatory functions of metabolic intermediates

Glycolytic remodeling in macrophages establishes a direct dialogue network between metabolism and immunity through the dynamic accumulation of intermediate metabolites. Intermediates from glycolysis and the TCA cycle (such as lactate, succinate, and itaconate) are not merely energy carriers but also function as signaling molecules directly participating in immune regulation (15).Beyond receptor-mediated signaling and allosteric regulation, these metabolites are increasingly recognized as precursors for novel post-translational modifications (PTMs), directly wiring metabolic activity to the functional reprogramming of the proteome and epigenome.

Lactate, the primary end-product of glycolysis, has been identified in *in vitro* and animal studies as a molecule with immunoregulatory capacity beyond its role as a metabolic substrate (90, 91). Emerging evidence suggests it may function as a signaling molecule, though the precise mechanisms *in vivo* remain to be fully elucidated.Upon mitochondrial entry, lactate activates the electron transport chain, thereby promoting oxidative phosphorylation while concurrently providing feedback inhibition of glycolysis (92, 93). Lactate directly modulates inflammatory signaling via the G protein-coupled receptor GPR81 (94), and the acidic microenvironment resulting from its accumulation inhibits TNF-α secretion (95) and suppresses NF-κB nuclear translocation, thereby reducing LPS-induced pro-inflammatory factor production (96).

A pivotal mechanism of lactate-mediated immune modulation is its epigenetic regulatory function through histone lactylation, a novel post-translational modification derived from the reaction between lactate and lysine residues. This PTM is catalyzed by acyltransferases like p300 and reversed by deacetylases including HDAC1-3 and SIRT1-3, providing enzymatic regulation for its dynamic nature. The extent of this modification is directly dependent on endogenous lactate generated by glycolysis (13). Lactate exhibits complex bidirectional regulation of immune function. During inflammatory stimulation, immune cells activate gluconeogenesis to promote glycogen deposition, providing sufficient ATP and biosynthetic precursors for pro-inflammatory responses (97) while significantly upregulating pro-inflammatory cytokine gene expression, including TNF-α and IL-6, in part through lactylation (98). Hyperactivated immune cells generate and secrete large amounts of lactate via glycolysis, leading to elevated local lactate concentrations that remodel immune cell functional phenotypes and can promote inflammatory responses (99).

Conversely, in a dynamic regulatory process termed the “lactate clock,” exogenous lactate supplementation under LPS stimulation paradoxically enhances PKM2 activity (100), which leads to the downregulation of inflammatory factors and upregulation of anti-inflammatory factors such as Arg1 and IL-10, thereby suppressing excessive inflammation and promoting tissue repair. This shift is facilitated by lactate activating M2-associated genes to suppress excessive inflammation and facilitate tissue repair. Given the central role of PKM2 in regulating glycolytic flux, rate, and lactate homeostasis, PKM2-mediated lactylation represents one of its potential anti-inflammatory mechanisms (13), underscoring the sophisticated crosstalk between metabolic and immune pathways in macrophage function.

Succinate accumulates significantly during bacterial infection due to metabolic reprogramming. Succinate is generated from TCA cycle intermediates catalyzed by succinate dehydrogenase (SDH), but in Mycobacterium tuberculosis (MTB)-infected macrophages, SDH expression decreases (101), leading to abnormal succinate buildup. On one hand, succinate stabilizes HIF-1α by inhibiting PHDs (102), directly enhances IL-1β transcriptional activity, and activates NLRP3 inflammasome to promote IL-1β maturation and release (15, 29) to drive burst secretion of pro-inflammatory factors (37, 103); on the other hand, SDH functional inhibition causes a second interruption of the TCA cycle, forcing increased glycolytic flux to maintain energy supply (101). Within mitochondria, oxidation of succinate by SDH triggers reverse electron transport, leading to elevated mitochondrial membrane potential and mitochondrial ROS (mROS) burst, thereby activating NLRP3 and AIM2 inflammasomes and promoting maturation/release of inflammatory factors including IL-1β, IL-18, and HMGB1 (37, 38). Additionally, succinate amplifies pro-inflammatory signals through protein succinylation (14) and cooperates with mitochondrial DNA (mtDNA) to activate damage-associated molecular pattern (DAMP) signaling, synergizing with TLRs to enhance host recognition and clearance of pathogens (29).

Itaconate serves as a key immunometabolite synthesized by immune-responsive gene 1 (IRG1), fulfilling dual roles in antimicrobial defense and inflammation resolution. It exerts potent anti-inflammatory effects through multiple mechanisms, including direct inhibition of NLRP3 inflammasome activation (104) and enhancement of antioxidant responses via modification of cysteine residues on the transcription factor nuclear factor erythroid 2-related factor 2 (Nrf2), thereby balancing inflammatory and oxidative stress pathways (105). Concurrently, itaconate possesses direct antibacterial activity, primarily through specific inhibition of bacterial isocitrate lyase (ICL) (106). The glyoxylate cycle (GAC) serves as an essential metabolic pathway for MTB survival under specific conditions, and ICL represents its key regulatory enzyme (107). By blocking ICL activity, itaconate effectively disrupts this critical bacterial metabolic pathway, compromising energy metabolism and restricting intracellular replication. The metabolic basis for itaconate-mediated defense involves tightly regulated biosynthesis during infection. Transcriptomic analyses reveal that MTB infection significantly upregulates IRG1 expression in both murine macrophages and lung tissues (101, 108), driving robust itaconate production as a tailored metabolic defense mechanism. This induction is accompanied by reduced expression of isocitrate dehydrogenase 2 (IDH2), which redirects citrate flux toward itaconate synthesis rather than the TCA cycle (107). This metabolic rewiring provides macrophages with an effective means to control early MTB proliferation while minimizing tissue damage from excessive inflammation, representing a finely tuned host adaptation that aligns metabolic reprogramming with antimicrobial requirements.

Other TCA cycle intermediates also participate in immunometabolic regulation. Fumarate, for instance, activates innate immune responses by inducing mitochondrial DNA release (109, 110), further highlighting the central role of metabolic intermediates in bridging redox balance with immune function. These coordinated mechanisms illustrate the sophisticated metabolic network through which macrophages deploy specific metabolites to simultaneously combat pathogens and regulate inflammatory responses.

Changes in metabolite levels also regulate gene expression through epigenetic mechanisms. Acetyl-CoA influences pro-inflammatory gene transcription by modulating histone acetylation levels (111), while accumulation of succinate and citrate in M1 macrophages stabilizes HIF-1α by inhibiting PHDs, promoting inflammation-related gene expression. This epigenetic regulation is particularly prominent in LPS-stimulated bone marrow-derived macrophages (BMDMs), where TCA cycle rupture leads to aberrant succinate and citrate accumulation (29), further amplifying inflammatory signals.

Expanding beyond these canonical signaling roles, recent research reveals that metabolic intermediates further refine macrophage responses by directly incorporating metabolic information into the proteomic landscape through a series of novel post-translational modifications (PTMs). For instance, in LPS-stimulated macrophages, the glycolytic enzyme GAPDH undergoes malonylation at lysine 213. This modification not only enhances its glycolytic activity but also liberates it from tumor necrosis factor–α (TNF-α) mRNA, thereby concurrently boosting metabolic flux and facilitating the translation of this key pro-inflammatory cytokine (112). This exemplifies a precise mechanism whereby a metabolic enzyme’s function is directly coupled to cytokine output via a metabolism-derived PTM. Furthermore, the immunometabolite itaconate can covalently modify cysteine residues on proteins, a process termed 2,3-dicarboxypropylation (2,3-DCP), which contributes to its anti-inflammatory effects through targets such as KEAP1, beyond its well-known role as an enzymatic inhibitor (113, 114). These findings establish metabolism-driven PTMs as a fundamental regulatory stratum, enabling metabolites to directly and rapidly reprogram macrophage function by altering protein activity and interactions, as summarized in **Table 1**.

**Table 1:** Key metabolites and regulatory molecules in macrophage glycolytic reprogramming and immune modulation.

Molecule	Proposed Mechanism(s)	Model System(s)	Representative References
Lactate	Acts as signaling molecule via GPR81; induces histone lactylation promoting M2 gene expression (Arg1, IL-10); suppresses NF-kB nuclear translocation; modulates PKM2 activity to promote M1-M2 transition	LPS-stimulated macrophages; murine models	(13, 94–96, 100)
Succinate	Stabilizes HIF-1α by inhibiting PHDs; enhances IL-1B transcription; drives mROS production via reverse electron transport at complex I; activates NLRP3 and AIM2 inflammasomes; induces protein succinylation	BMDMs;MTB-infe cted macrophages; murine sepsis models	(14, 29, 37, 38), (101–103)
itaconate	Synthesized by IRG1; inhibits NLRP3 inflammasome; activates Nrf2 antioxidant pathway via cysteine modification; inhibits bacterial isocitrate lyase (ICL), disrupting glyoxylate cycle.	MTB-infected macrophages; murine models; LPS-stimulated BMDMs	(104–106, 108, 113, 114)
NAD'/NA DH	Low NAD'/NADH ratio promotes NLRP3 inflammasome activation via HIF-10-succinate axis; suppresses MAVS-IRF3 mediated type I IFN production; restoring NAD' enhances STING-dependent IFN-B expression; regulates autophagy via AMPK/mTOR	S.Typhimurium-in fected macrophages; NAD' precursor supplementation models;Ndufs4 KO mice	(28, 30–32, 115, 116)
Pyruvate	Diverted to lactate via LDHA in aerobic glycolysis; accumulation induces expression of Salmonella virulence island SPI-2; serves as carbon source for intracellular bacterial replication.	Salmonella-infect ed macrophages	(10, 70, 117)
PKM2	Conformational switching between tetramer (OXPHOS) and dimer (Warburg effect); nuclear dimer acts as HIF-1a co-activator enhancing IL-1B transcription; regulates NLRP3/AIM2	LPS-stimulated macrophages; murine sepsis models; PKM2 KO mice	(36–38, 41, 43, 45, 46)
	inflammasome activation; TEPP-46 stabilizes tetramer to suppress inflammation		
GLUT1	Initiates glycolytic reprogramming by enhancing glucose uptake; triggers "glucose-insulin-IL-18" positive feedback loop; downregulation by Salmonella induces host cell apoptosis.	LPS-stimulated macrophages; insulin signaling models	(47, 48, 118)
SIRTS	Desuccinylates PDHA1 to restrain Warburg effect; promotes M2 polarization via glutaminolysis; deficiency exacerbates IL-1B production	Eosinophilic nasal polyps; clear cell renal cell carcinoma; SIRTS KO models	(49–51)
CAMP	Killed bacteria release CAMP, activating AMPK; inhibits mTORC1, promoting metabolic recycling; enhances macrophage survival and antioxidant responses.	Macrophages phagocytosing killed vs. viable bacteria	(119)

The preceding discussion has highlighted the central role of metabolic intermediates and regulatory molecules in orchestrating macrophage immune functions during bacterial infection. To provide readers with a concise overview, we have summarized key metabolites and molecular regulators discussed in this review in **Table 1**. For each molecule, we outline its proposed immunometabolic mechanisms, the experimental model systems in which these mechanisms have been elucidated, and the primary supporting references. This table is not intended to be exhaustive but rather to capture the most extensively studied and mechanistically well-characterized molecules that illustrate the intricate crosstalk between glycolysis and immunity. As will become evident in subsequent sections, several of these metabolic nodes are also exploited by bacterial pathogens for immune evasion, underscoring their dual significance in host-pathogen interactions.

### 3.2 Bacterial hijacking of glycolytic metabolism and immune evasion

Macrophage glycolytic remodeling represents a critical regulatory node in host antibacterial immunity, establishing a dynamic metabolic battlefield where the outcomes of infection are determined by the interplay between host defense and pathogen evasion strategies (120). While the host leverages metabolic reprogramming to launch antimicrobial attacks, diverse bacterial pathogens have evolved to exploit, subvert, or even suppress these very metabolic pathways to facilitate their survival and replication.

The strategic manipulation of host metabolism by bacteria can lead to opposing outcomes. *Mycobacterium tuberculosis (MTB)* infection triggers a host-protective immunometabolic response by driving macrophages toward aerobic glycolysis. This shift, characterized by the upregulation of key glycolytic enzymes and downregulation of TCA cycle and OXPHOS components, is essential for inducing a potent pro-inflammatory response, including the enhancement of IL-1β secretion, which ultimately limits bacterial intracellular survival (9, 121). In stark contrast, *Legionella pneumophila* actively hijacks host metabolism to establish a replication-permissive niche. Via its type IV secretion system (T4SS), the bacterium delivers effector proteins such as MitF, which induces mitochondrial fragmentation and abruptly halts mitochondrial respiration. Concurrently, the infection upregulates host glycolysis, collectively forcing the macrophage into a Warburg-like state that is essential for its intracellular replication (122).In contrast, *Francisella tularensis* adopts the opposite approach by inhibiting macrophage glycolysis, forcing metabolic reversion to OXPHOS to maintain host cell viability and prolong its replication cycle. Additionally, *Francisella tularensis* infection suppresses macrophage lactate production, HIF-1α stability, and aerobic glycolysis, further promoting early bacterial replication (123).

*Salmonella* employs particularly sophisticated metabolic hijacking mechanisms. *Salmonella enterica* serovar Typhimurium inhibits the host serine synthesis pathway via type III secretion system (T3SS) effector proteins, causing 3-phosphoglycerate accumulation that bacteria directly utilize as a carbon source for replication. Infection upregulates host glycolysis while reducing serine synthesis, leading to pyruvate and lactate accumulation; these intermediates induce expression of virulence island SPI-2, enhancing bacterial intracellular virulence. The SPI-1 effector protein SopE2 further drives proliferation by promoting serine synthesis (10). Meanwhile, *Salmonella* Enteritidis promotes host lactate generation and exploits the T3SS2 effector SteE to induce M2 macrophage polarization, thereby suppressing inflammation and creating an immunosuppressive environment favorable for bacterial proliferation (124). *Salmonella* infection also promotes M2 polarization by increasing glucose utilization, hijacking glycolytic intermediate pyruvate to further activate virulence gene expression and enhance invasive capacity (117).

Other pathogens employ alternative strategies to hijack host metabolic networks. *Candida* forces macrophage energy exhaustion through competitive glucose consumption, activating AMPK-Bcl-2-associated X protein/Bcl-2 homologous antagonist killer (Bax/Bak)-dependent apoptosis (125), while *Yersinia pseudotuberculosis* conversely activates AMPK to inhibit apoptosis, maintaining host cell survival to promote intracellular replication (126). Other bacteria sustain energy supply by directly acquiring glycolytic intermediates or inhibiting OXPHOS (127).

Pathogens also strategically manipulate host cell death pathways. *Salmonella Typhimurium* downregulates GLUT1 to inhibit glucose uptake, causing ATP depletion and AMPK activation, this phosphorylates pro-apoptotic protein Bcl-2-associated death promoter (BAD), inducing mitochondrial membrane permeability transition pore opening and caspase-9/3-dependent apoptosis, accelerating host cell death (118). Concurrently, *Salmonella Typhimurium* activates NLRP3 inflammasome-mediated pyroptosis by disrupting glycolytic flux (128); this mechanism relates to hexokinase activity regulation bacterial peptidoglycan-derived N-acetylglucosamine inhibits hexokinase activity, causing its dissociation from mitochondria and NLRP3 inflammasome activation (129). Additionally, *Salmonella Typhi* inhibits STAT1 activation and IL-12 secretion by deleting SPI2 effectors targeting NF-κB and the SPI1 effector SarA, weakening host immune responses to establish persistent infection (130).

Conversely, *Shigella flexneri* secretes effector protein OspC3, which blocks activation of caspase-4/11 by modifying R314/310 residues, inhibiting cleavage of gasdermin D (GSDMD), thereby evading caspase-4/11 GSDMD-mediated host immunity against intracellular Gram-negative bacteria (131). Dynamic changes in the metabolic microenvironment directly shape cell death outcomes: under high-glucose conditions, lactate accumulation triggers GSDMD-dependent pyroptosis via ubiquitin-mediated HIF-1α degradation (132); the vascular endothelial growth factor receptor 3 (VEGFR3)-AMPK signaling axis promotes apoptosis via BAD phosphorylation while suppressing caspase-1-mediated inflammasome activation; its regulatory direction is closely related to the dynamic balance of glycolytic metabolites (12).

While pathogens actively manipulate host metabolism for immune evasion, recent evidence reveals that macrophages are not merely passive victims but can strategically metabolize phagocytosed bacteria to fuel their own immunometabolic programs. A seminal study demonstrates that macrophages phagocytose and degrade bacteria, repurposing microbial-derived nutrients including amino acids and carbon skeletons into central metabolic pathways such as glutathione and itaconate biosynthesis (119). This metabolic recycling is regulated by the nutrient-sensing mTORC1 pathway and is highly dependent on bacterial viability. Notably, killed bacteria enriched in cyclic AMP (cAMP) sustain cellular AMP pools and activate AMPK, which inhibits mTORC1 and promotes metabolic recycling. This process enhances macrophage survival and antioxidant responses while reducing ROS and IL-1β secretion, contrasting with the pro-inflammatory outcomes triggered by viable bacteria. This viability-sensing mechanism enables macrophages to dynamically adjust their immunometabolic responses based on the nature of the ingested cargo, representing a sophisticated host strategy to counteract bacterial metabolic hijacking.

In summary, macrophage glycolytic remodeling exhibits bidirectional roles in bacterial infection: the host activates antibacterial immunity through metabolic reprogramming, while pathogens reshape immune response landscapes by hijacking key nodes of glycolysis, OXPHOS, or the TCA cycle, ultimately achieving immune evasion and intracellular survival. Furthermore, macrophages can actively counteract this hijacking by metabolizing killed bacteria to support survival and resolution pathways. Pathogenic strategies for immune evasion extend beyond the manipulation of metabolic flux to include the direct sabotage of the host’s metabolic signaling apparatus. A striking example is Staphylococcus aureus, which subverts innate immunity by suppressing the palmitoylation of STING (Stimulator of Interferon Genes) in host macrophages (133). Palmitoylation is a lipid modification indispensable for STING’s activation and its trafficking from the endoplasmic reticulum to the Golgi apparatus, a critical step for initiating type I interferon production. By impairing this specific PTM, S. aureus effectively blunts a cornerstone of the host’s antibacterial defense, unveiling a sophisticated evasion mechanism that operates at the level of post-translational control.

Targeting critical hubs of metabolic pathways (for example, restoring NF-κB activity, modulating NAD^+^/NADH ratio, or enhancing pyroptotic signals) can reverse pathogen metabolic hijacking, providing new directions for anti-infection therapy. Future studies should integrate single-cell metabolomics with spatiotemporal intervention technologies to dissect the heterogeneity of metabolic networks and develop dual-target inhibitors to counter pathogen evolution, ultimately realizing a “precision metabolic immunology” therapeutic paradigm.

### 3.3 Coupling of M1/M2 polarization and metabolic reprogramming in immune regulation

The intricate coupling between macrophage phenotypic polarization and metabolic reprogramming represents a fundamental mechanism in immune regulation, with dynamic temporal progression throughout infection. M1 macrophages’ pro-inflammatory functions are characterized by aerobic glycolysis as the core metabolic feature, manifested by significant enhancement of glycolysis and the pentose phosphate pathway (PPP), accompanied by TCA cycle truncation, mitochondrial respiratory chain inhibition, and excessive ROS accumulation (39, 134). During early infection stages, pathogen-associated molecular patterns (PAMPs) drive expression of key glycolytic enzymes including GLUT1 and HK2 via TLR4/NF-κB pathways, promoting M1 polarization and pro-inflammatory cytokine secretion while effectively controlling bacterial load through rapid ATP generation (135, 136). Microenvironmental factors such as interferon gamma (IFN-γ) and LPS stabilize this M1 phenotype through activation of PKM2. Under glycolysis-dominated metabolism, rapidly generated ATP supports phagocytosis and burst secretion of pro-inflammatory factors including IL-1β and TNF-α (39), while the PPP provides dual support for antioxidant defense and ROS burst by generating ribose for nucleotide synthesis and NADPH (134). At this stage, M2-related genes (such as Arg1) maintain low expression levels (137), indicating early coupling between metabolic patterns and immune responses. Mitochondrial ETC abnormalities resulting from functional inhibition lead to ROS bursts that activate the NLRP3 inflammasome and induce oxidative stress damage to cellular components, further amplifying inflammatory cascades. Experimental evidence confirms that inhibiting mitochondrial ETC mimics LPS effects, driving macrophage polarization toward the M1 phenotype (40), highlighting the central role of metabolic remodeling in pro-inflammatory polarization.

The stability of M1 phenotype involves self-reinforcing metabolic-inflammatory circuits where LPS-induced PKM2 dimerization facilitates nuclear translocation, serving as HIF-1α coactivator at the IL-1β promoter to amplify pro-inflammatory gene transcription (41, 138). However, as infection progresses, multiple converging signals orchestrate the transition toward M2 dominance. Glucose depletion activates AMPK, shifting metabolism from glycolysis to fatty acid oxidation by suppressing mTORC1 signaling and inducing M1-to-M2 phenotypic conversion (12). This metabolic reconfiguration receives reinforcement from changing cytokine milieus, particularly IL-4 and IL-13, which further downregulate glycolytic flux and restore mitochondrial OXPHOS by inhibiting mTOR signaling (134), leading to reduced HIF-1α levels (139). M2 macrophages depend on FAO and OXPHOS for energy, with metabolic features highly adapted to anti-inflammatory and tissue repair functions (40, 140). Cytokines such as IL-4/IL-13 enhance mitochondrial respiratory chain activity to promote M2 polarization (135). An intact TCA cycle coupled with OXPHOS generates ATP, supporting Arg1 and procollagen production to promote tissue remodeling and repair (39). This metabolic transition marks the shift from antibacterial defense to tissue repair functions, though it may occur alongside rising bacterial loads (136, 141).

The dynamic transition between these states is precisely regulated by multiple molecular switches. Peroxiredoxin 3 (PRDX3) accelerates M1-to-M2 transition by restoring TCA cycle function (142), while PKM2 conformational changes coordinate phenotypic switching by regulating lactate metabolism (13). PKM2 exhibits dual regulatory capacity in polarization dynamics: its conformational changes can both attenuate M1 inflammation and enhance M2 anti-inflammatory functions (36). Inhibition of PKM2 nuclear translocation may suppress pro-inflammatory phenotypic polarization of macrophages, preventing further inflammatory infiltration. In LPS-induced macrophages, exogenous lactate increases mediate PKM2 activity, initiating the transcriptional activation program of M2-related genes through epigenetic regulatory mechanisms in terminally differentiated M1 macrophages, while significantly downregulating the expression levels of M1 macrophage-specific markers, thereby promoting the M1 to M2 transition (100). Thus, PKM2 plays a crucial role in macrophage polarization and represents a potential key target for inhibiting inflammatory responses. The Notch signaling pathway dynamically regulates polarization thresholds by balancing glycolysis and OXPHOS metabolic fluxes (143). Enhancing mitochondrial respiration in M1 cells promotes M2 marker expression (40), demonstrating the metabolic plasticity underlying polarization dynamics.

The metabolic basis of polarization is further illuminated by bacterial viability sensing. Phagocytosis of killed bacteria promotes an anti-inflammatory metabolic signature characterized by enhanced itaconate and glutathione production, reduced ROS, and decreased IL-1β secretion features consistent with an M2-like phenotype. In contrast, viable bacteria sustain a pro-inflammatory, M1-like state with elevated ROS and IL-1β release (119). This viability-sensing mechanism, mediated through bacterial cAMP-AMP-AMPK signaling that inhibits mTORC1 and redirects metabolism toward antioxidant pathways, enables macrophages to dynamically adjust their polarization state based on the ingested cargo. Carboxykinase-like protein (CARKL), highly expressed in M2 cells, suppresses pro-inflammatory signals by limiting PPP flux (134). OXPHOS integrity is crucial for M2 function, as inhibition with oligomycin A or carbonyl cyanide-p-trifluoromethoxyphenylhydrazone (FCCP) significantly impairs expression of M2 markers including Arg1. Mitochondrial function intervention experiments further confirm that inhibiting OXPHOS in M2 cells reverses their anti-inflammatory properties (40).

**Figure 2:**
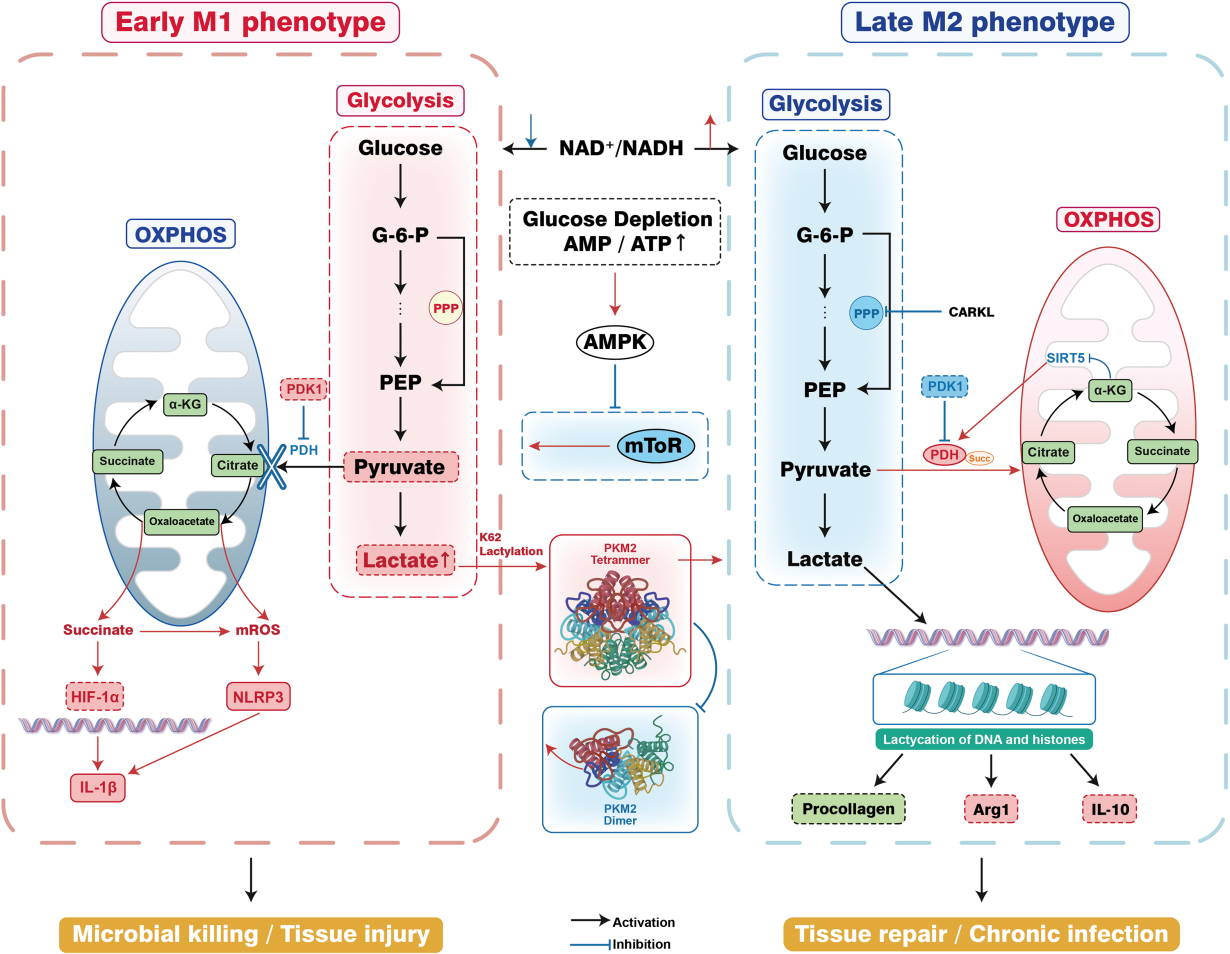
Glycolysis orchestrates macrophage M1/M2 polarization through an immunometabolic network during early and late stages of infection. This figure depicts the temporal immunometabolic switch governing macrophage function. In the early phase, glycolytic flux is enhanced and the TCA cycle is interrupted, leading to succinate accumulation which drives HIF-1α stabilization, mROS production, and IL-1β expression for effective microbial killing. As infection progresses and glucose is depleted, the rising AMP/ATP ratio activates AMPK. Concurrent lactate accumulation stabilizes the PKM2 tetramer, facilitating a metabolic and functional transition. In the late M2 phenotype, this lactate installs a reparative epigenetic landscape via histone lactylation, promoting the expression of anti-inflammatory mediators (Arg1, IL-10) and procollagen to direct tissue repair.

However, imbalanced metabolic-immune coupling may lead to pathological consequences. Sustained glycolytic activation weakens host defense by inhibiting NF-κB nuclear translocation (96), while OXPHOS functional defects hinder tissue repair processes (144). Although targeting metabolic nodes such as activating AMPK or inhibiting HIF-1α can directionally regulate polarization (12), specific molecular mechanisms governing metabolic switching in late infection remain incompletely understood. Key knowledge gaps persist regarding the roles of enzymes such as IDH2 and SDH in metabolic homeostasis, and molecular pathways for mitochondrial functional recovery require further exploration to fully elucidate the sophisticated interplay between metabolism and immunity in macrophage polarization(**Figure 2**).

### 3.4 Metabolic regulation of autophagy in infection immunity and pathological transformation

As a key effector mechanism bridging cellular metabolism and immune clearance, autophagy is dynamically regulated by the immunometabolic state of macrophages during bacterial infection. Under physiological conditions, activated AMPK and glycolytic reprogramming synergistically enhance autophagosome formation, supporting the clearance of intracellular pathogens like Salmonella while suppressing excessive pyroptosis (12). This metabolic-autophagic coupling provides a crucial homeostatic mechanism for maintaining immune balance during early infection.

However, pathogens and pathological metabolic microenvironments can subvert this protective mechanism into a driver of immunopathology. In diabetic periodontitis models, hyperglycemia inhibits autophagic flux through the mTOR-ULK1 pathway, leading to mitochondrial ROS accumulation and promoting Porphyromonas gingivalis-induced NLRP3 inflammasome activation and pyroptosis (140). The resulting gasdermin D-mediated cell death and IL-1β secretion exacerbate alveolar bone destruction, illustrating how metabolic disruption transforms autophagy from a defense mechanism into an immunopathological conduit. Therapeutic interventions targeting this network including zingerone that restores autophagic activity and the NLRP3 inhibitor MCC950 that blocks pyroptotic signaling demonstrate that modulating the autophagy-metabolism-inflammation axis can effectively mitigate infection-related tissue damage in metabolically compromised environments (145).

Notably, the NAD+/NADH ratio emerges as a critical regulator of autophagic integrity during infection. A decreased NAD+/NADH ratio impairs the AMPK/mTOR signaling network, disrupting the homeostatic regulation of autophagy and contributing to immune defense collapse (115, 116). This metabolic-sensitive regulatory node directly connects mitochondrial redox status to autophagic function, providing a mechanistic explanation for how bacterial-induced metabolic reprogramming can compromise this essential clearance pathway.

The dual role of autophagy in infection immunity balancing “pro-survival” clearance functions against “pro-death” inflammatory pathways is therefore strictly determined by the metabolic context. Understanding how bacterial infections and metabolic disorders reshape this balance offers new therapeutic opportunities for targeting the autophagy-metabolism-inflammation network to preserve immune defense while minimizing pathological damage in complex infection scenarios.

## 4 Glycometabolism in polymicrobial bacterial infection

Bacterial glycometabolism exhibits significant differences between monomicrobial and polymicrobial infections, with interaction patterns (synergistic or antagonistic) jointly regulated by pathogen species, metabolic microenvironment, and host immune status. Research indicates that in mixed infections, bacteria can remodel infection progression through the following mechanisms:

### 4.1 Biofilm synergy and antibiotic resistance

In Staphylococcus aureus and Pseudomonas aeruginosa co-infection, dynamic regulation of glycometabolism is a key mechanism for synergistic formation of stratified biofilms and mediation of antibiotic resistance (146). When Pseudomonas aeruginosa occupies the biofilm surface layer, it rapidly consumes environmental glucose through highly active glycolysis, driving alginate synthesis this process depends on accumulation of the glycolytic intermediate uridine diphosphate glucose (UDP-glucose). Alginate forms a dense extracellular matrix that physically impedes antibiotic penetration, and its synthesis itself relies on sustained glycolytic flux. Simultaneously, surface-layer P. aeruginosa suppresses virulence factor expression in underlying S. aureus via quorum-sensing molecules (such as C4-HSL); this regulation likely extends to glycometabolic pathways, restricting glucose uptake or glycolytic efficiency in S.aureus, forcing it into a metabolically quiescent persister state, thereby enhancing tolerance to vancomycin. On the other hand, metabolic adaptation of S.aureus counteracts P. aeruginosa: although P. aeruginosa occupies the surface niche through glycometabolic superiority, phenol-soluble modulins (PSMs) secreted by underlying S.aureus interfere with P. aeruginosa energy metabolism, inhibiting OXPHOS or key glycolytic enzyme activity, reducing ATP generation. This metabolic interference not only directly suppresses P. aeruginosa growth but may also enhance killing efficacy of ciprofloxacin and aminoglycosides by disrupting energy homeostasis, as bactericidal efficiency of these antibiotics is closely linked to bacterial metabolic activity. Through competition for glycometabolic resources and cross-regulation of metabolites, the two bacteria form a complex network of both antagonism and symbiosis within biofilms, ultimately synergistically amplifying antibiotic resistance.

### 4.2 Metabolic competition and microenvironmental regulation

High-glucose microenvironments critically regulate outcomes in mixed infections. Under normal conditions, *P. aeruginosa* competitively inhibits S*. aureus* growth via siderophores; however, hyperglycemia activates glucose transporters and key glycolytic enzymes in S*. aureus*, significantly enhancing its glycolytic flux, reversing metabolic disadvantage and driving deeper tissue invasion in co-infection (147). Similarly, *Staphylococcus epidermidis* and *Staphylococcus hominis* inhibit S*. aureus* biofilm formation by secreting antimicrobial peptides (such as lantibiotics), while the prebiotic fructooligosaccharide further strengthens colonization advantage of *S. epidermidis*, enhancing suppression of S*. aureus* biofilm formation (148, 149).

### 4.3 Phenotypic switching and immune evasion

Mixed infections may induce adaptive phenotypic changes in pathogens. For example, *S. aureus* forms small-colony variants (SCVs) when coexisting with *P. aeruginosa*, exhibiting reduced metabolic activity but significantly enhanced antibiotic tolerance. This phenotypic switch is closely linked to host immunosuppression: SCVs evade macrophage phagocytic killing by downregulating pro-inflammatory factors (such as TNF-α) while suppressing neutrophil extracellular trap (NETs) formation, exacerbating risks of chronic infection (146).

## 5 Conclusion and future perspectives

The dynamic coupling between macrophage glycolytic remodeling and immune responses represents a core regulatory mechanism in host defense against bacterial infection. During bacterial infection, macrophages significantly upregulate glycolytic rate-limiting enzymes including GLUT1, HK2, PKM2, and LDHA, while suppressing oxidative phosphorylation through HIF-1α, NF-κB, and PI3K/Akt/mTOR signaling networks. These changes collectively drive metabolic flux toward aerobic glycolysis (11, 37).This metabolic remodeling provides energetic support for pro-inflammatory cytokine secretion such as IL-1β and TNF-α and for phagocytic function through rapid ATP and NADPH generation. At the same time, it modulates immune response intensity via the signaling functions of metabolic intermediates. Notably, glycolytic reprogramming does not occur in isolation but operates within a broader metabolic network. During M1 polarization, for instance, glutamine catabolism anaplerotically replenishes the TCA cycle to support aspartate and itaconate synthesis (150), indicating that cross-talk between amino acid metabolism and glycolysis contributes significantly to immune outcomes.

The strategies employed by pathogens to hijack key metabolic nodes for immune evasion further illustrate the complexity of metabolic-immune networks. Salmonella utilizes T3SS effector proteins to divert pyruvate for activating virulence gene expression (10).Mycobacterium tuberculosis induces glycolysis to enhance IL-1β secretion while simultaneously inhibiting OXPHOS to attenuate mitochondrial anti-infection functions (9). These mechanisms demonstrate that metabolic remodeling serves both as a driver of host antibacterial defense and as a target for pathogen immune evasion.

Despite considerable progress in understanding macrophage immunometabolism, several challenges and knowledge gaps merit further discussion. First, the literature contains apparent contradictions regarding the role of specific metabolic pathways in host defense. Aerobic glycolysis, for instance, is widely recognized as a metabolic hallmark of M1 polarization and is considered essential for bacterial clearance (36, 39). However, emerging evidence suggests that excessive or sustained glycolytic activation may paradoxically impair host defense by inhibiting NF-κB nuclear translocation and promoting an immunosuppressive microenvironment (96, 136). Similarly, itaconate has been reported to exert both anti-inflammatory (104, 105) and pro-inflammatory effects (108), depending on cellular context, infection stage, and local metabolite concentrations.

The context-dependent nature of metabolic interventions poses significant challenges for therapeutic development. The same metabolic manipulation may yield divergent outcomes based on infection stage, pathogen type, and tissue microenvironment. AMPK activation, for example, promotes bacterial clearance in certain experimental models (12) but has been associated with enhanced pathogen survival in others (126). This variability underscores the need for spatiotemporally precise intervention strategies rather than broad metabolic modulation.

The safety profiles of metabolism-targeting agents remain inadequately characterized. While small-molecule compounds such as TEPP-46 (a PKM2 activator) have demonstrated efficacy in murine sepsis models (36), critical questions regarding bioavailability, tissue distribution, and long-term effects on systemic metabolism remain unanswered. Notably, TEPP-46 has been shown to enhance PKR phosphorylation under certain experimental conditions, raising the possibility of unintended NLRP3 inflammasome activation. Celastrol, which inhibits PKM2 nuclear translocation, presents different translational hurdles, including poor aqueous solubility, limited oral bioavailability, and potential hepatotoxicity at therapeutic doses (43). Even for well-validated targets such as GPR91 in inflammatory bowel disease (29), the ubiquitous expression of this receptor raises concerns about off-target effects, highlighting the need for tissue-specific delivery systems.

Species differences in immunometabolic pathways complicate the translation of findings from murine models to humans. Macrophage metabolic responses to LPS and bacterial infection differ between mice and humans with respect to glycolytic flux, itaconate production, and inflammatory cytokine profiles (101, 108). These interspecies variations underscore the importance of validating key findings in human primary macrophages and clinical samples.

Clinical evidence linking macrophage metabolic reprogramming to human infectious disease outcomes remains limited. Current knowledge derives predominantly from *in vitro* studies and animal models, with few prospective clinical investigations examining metabolic signatures in patient cohorts or correlating these signatures with clinical endpoints such as mortality, organ dysfunction, or infection clearance. Whether targeting macrophage metabolism can improve these outcomes in human sepsis or chronic infections remains to be established.

The concept of targeting metabolic-immune nodes, termed by some as metabolic immune engineering, has garnered considerable interest, and preclinical data provide grounds for optimism. TEPP-46 stabilizes the tetrameric conformation of PKM2 and has been shown to suppress inflammasome activation and improve survival in murine sepsis models. However, translation of these findings to clinical applications requires addressing several unresolved questions. These include whether TEPP-46 achieves therapeutic concentrations in target cells following systemic administration, its tissue distribution profile, and the potential consequences of enhanced PKR phosphorylation observed under certain experimental conditions, which could theoretically trigger the very inflammasome activation the compound is intended to suppress. The long-term effects of PKM2 modulation on systemic glucose homeostasis remain unexplored, a consideration relevant not only to TEPP-46 but to any therapeutic agent targeting core metabolic pathways (36).Similar considerations apply to other candidate interventions. Inhibition of the succinate-GPR91 axis has been demonstrated to attenuate pathology in murine models of inflammatory bowel disease (29). Yet GPR91 is expressed ubiquitously across multiple tissues, raising concerns about off-target effects following systemic administration. Development of gut-specific delivery systems may be necessary to realize the therapeutic potential of this approach while minimizing systemic exposure. Celastrol, a natural compound that inhibits PKM2 nuclear translocation, reduces cytokine storm and multi-organ injury in sepsis models (43). Its clinical development is constrained by biopharmaceutical limitations including poor aqueous solubility, limited oral bioavailability, and potential hepatotoxicity at therapeutic doses. Formulation optimization may address some of these challenges, though this would require additional development time and resources.

Even strategies targeting post-translational modifications, theoretically a more specific approach, face significant hurdles. H-151, which inhibits STING palmitoylation, has shown efficacy in curbing STING-driven inflammatory pathology in preclinical models (151). However, palmitoylation regulates numerous proteins beyond STING. The selectivity of such inhibitors and the potential consequences of inhibiting a modification with broad biological functions require careful investigation.

Future research should prioritize several directions. Longitudinal metabolic profiling in human infections could help identify context-specific metabolic signatures amenable to therapeutic targeting. Development of cell-type-specific and temporally controlled metabolic interventions may minimize off-target effects. Rigorous preclinical safety and toxicity evaluation of candidate metabolic drugs should precede clinical translation. Integration of multi-omics approaches may help resolve species differences and identify evolutionarily conserved therapeutic targets. Early-phase clinical trials with well-defined metabolic and immunological endpoints are essential to bridge the current translational gap.

Collectively, these emerging strategies, though still in early stages of development, aim to reverse pathological metabolic reprogramming with greater specificity. By integrating rigorous preclinical validation with cautious clinical translation, they hold the potential to synergistically enhance antibiotic efficacy and recalibrate dysregulated immune responses in infectious and inflammatory diseases.
